# Effectiveness of BMMSC and Dienogest on IL-1β, IL-8 Expression and Lesion Area in Endometriosis Mice

**DOI:** 10.5935/1518-0557.20260001

**Published:** 2026

**Authors:** Rista Silvana, Yusuf Effendi, Rizani Amran, Widjiati Widjiati

**Affiliations:** 1 Department of Obstetrics and Gynecology, Faculty of Medicine, Universitas Sriwijaya / Dr. Mohammad Hoesin General Hospital, Palembang, Indonesia; 2 Faculty of Medicine, Universitas Muhammadiyah Palembang, Palembang, Indonesia; 3 Faculty of Veterinary Medicine, Universitas Airlangga, Surabaya, Indonesia

**Keywords:** bone marrow mesenchymal stem cells, Dienogest, endometriosis, IL-1β, IL-8

## Abstract

**Objective:**

Endometriosis is a chronic estrogen-dependent inflammatory disease that impairs fertility and quality of life. Dienogest, the current standard therapy, provides symptom relief but does not correct immune imbalance or prevent recurrence. Bone marrow-derived mesenchymal stem cells (BMMSCs), with strong immunomodulatory potential, may represent a disease-modifying alternative. This study evaluated the effects of BMMSCs, Dienogest, and their combination on IL-1β and IL-8 expression and lesion size in a murine endometriosis model.

**Methods:**

The experiment was conducted between August and December 2024 using 32 female mice, randomly assigned into four groups: untreated endometriosis controls (K+), BMMSC monotherapy (P1), BMMSC combined with Dienogest (P2), and Dienogest monotherapy (P3). Mice were injected intraperitoneally with endometrial fragments (200 µL) to become endometriosis models. On day 15, the models received mesenchymal stem cells. Sample collection was on day 29. Cytokine expression (IL-1β and IL-8) was assessed immunohistochemically, while lesion dimensions were analyzed using ImageJ v1.54 (NIH, USA) software. Immunofluorescence confirmed the BMMSC phenotype (CD73+, CD90+, CD105+, CD45-). Statistical analysis used one-way ANOVA and Kruskal-Wallis with Bonferroni correction.

**Results:**

BMMSC monotherapy resulted in the most pronounced suppression of IL-1β and IL-8 expression, alongside a significant reduction in lesion size (*p*<0.001). All treatment groups differed significantly from the control group.

**Conclusions:**

BMMSC monotherapy in endometriosis mice models markedly reduced IL-1β and IL-8 expression, decreased lesion size, and demonstrated more robust disease-modifying activity compared to Dienogest or combination therapy.

## INTRODUCTION

A growing number of studies revealed that 10% of women have been affected by endometriosis, a chronic gynecological disorder. It is widely considered a major cause of dysmenorrhea, pelvic pain, and infertility (Barnhart *et al.*, 2002; Omland *et al.*, 2005; Gupta *et al.*, 2008). Currently, it is not only a hormone-dependent disease but also a persistent inflammatory condition characterized by immune dysregulation and an altered peritoneal microenvironment (Harada *et al.*, 1999; Gupta *et al.*, 2008).

The disorder negatively impacts fertility, especially in women undergoing assisted reproductive technologies. Clinical and experimental studies demonstrate that endometriosis disrupts ovarian, tubal, and uterine physiology, resulting in poor oocyte quality, impaired fertilization, and decreased implantation rates (Barnhart *et al.*, 2002; Omland *et al.*, 2005). Inflammatory activation within the peritoneal cavity promotes recruitment of macrophages and excessive secretion of cytokines such as IL-1β, IL-8, TNF-α, and VEGF (Iwabe *et al.*, 1998; von Wolff *et al.*, 1999; Mahnke *et al.*, 2000; Witz, 2000; Ueda *et al.*, 2002). These proinflammatory mediators enhance angiogenesis, fibrotic remodeling, granulosa cell apoptosis, and oxidative stress, ultimately compromising reproductive potential (Khan *et al.*, 2014; Da Broi *et al.*, 2016; Sanchez *et al.*, 2016; 2017; Giacomini *et al.*, 2017; Wu *et al.*, 2017).

Elevated levels of IL-1β, IL-8, and TNF-α in follicular and peritoneal fluid have been strongly associated with impaired oocyte competence, reduced fertilization, and suboptimal embryo development (Khan *et al.*, 2014; Da Broi *et al.*, 2016; Giacomini *et al.*, 2017; Sanchez *et al.*, 2017; Wu *et al.*, 2017). Persistent inflammation drives lesion proliferation and recurrence, even after conventional therapy (Santamaria *et al.*, 2012; Becker *et al.*, 2017; Viganò *et al.*, 2018).

Current management relies mainly on hormonal therapy. Dienogest, a fourth-generation progestin, provides symptom relief by suppressing ovulation and inducing endometrial atrophy (Andres *et al.*, 2015; Bedaiwy *et al.*, 2017; Wu *et al.*, 2022; Muzii *et al.*, 2023). However, it does not fully resolve immune imbalance, is associated with side effects, and recurrence is common upon discontinuation (Andres *et al.*, 2015; Becker *et al.*, 2017; Bedaiwy *et al.*, 2017; Szukiewicz, 2022; Wu *et al.*, 2022; Muzii *et al.*, 2023; Piriyev *et al.*, 2025). Meta-analyses confirm that while Dienogest alleviates symptoms, it does not prevent disease progression (Andres *et al.*, 2015; Wu *et al.*, 2022; Muzii *et al.*, 2023).

Scholars have paid close attention to Mesenchymal stem cells (MSCs) because they contain immunomodulatory and regenerative characteristics (Uccelli *et al.*, 2008). Bone marrow-derived MSCs (BMMSCs) are well characterized and fulfill ISCT minimal criteria: adherence to plastic, fibroblast-like morphology, positive for CD73, CD90, and CD105, and negative for CD45 (Dominici *et al.*, 2006; Ghaneialvar *et al.*, 2018; Schmelzer *et al.*, 2019). These cells exert their effects via paracrine mechanisms, including secretion of cytokines, growth factors, and extracellular vesicles (Dominici *et al.*, 2006; Uccelli *et al.*, 2008; Chen *et al.*, 2018; Ghaneialvar *et al.*, 2018; Schmelzer *et al.*, 2019; Bian *et al.*, 2022; Műzes & Sipos, 2022; Kulesza *et al.*, 2023).

In reproductive models, BMMSCs have been shown to reduce granulosa cell apoptosis, enhance ovarian function, and modulate immune responses (Chen *et al.*, 2018; Rajabzadeh *et al.*, 2019; Bozorgmehr *et al.*, 2020; Na & Kim, 2020; Liao *et al.*, 2021; Cui & Jing, 2024; Kavaldzhieva *et al.*, 2025). However, direct comparisons between BMMSC therapy and standard hormonal treatments for endometriosis remain scarce (Vernon & Wilson, 1985; Grümmer, 2006; Bedaiwy *et al.*, 2017). Considering the central roles of IL-1β and IL-8 in lesion maintenance and infertility (Iwabe *et al.*, 1998; von Wolff *et al.*, 1999; Mahnke *et al.*, 2000; Witz, 2000; Ueda *et al.*, 2002; Khan *et al.*, 2014; Da Broi *et al.*, 2016; Giacomini *et al.*, 2017; Sanchez *et al.*, 2017; Wu *et al.*, 2017), evaluating BMMSCs against Dienogest is crucial.

The novelty of this study lies in its direct head-to-head comparison of BMMSCs, Dienogest, and their combination in a murine endometriosis model. Previous studies have demonstrated that MSCs possess immunomodulatory and regenerative potential in endometriosis by reducing inflammatory cytokines, inhibiting fibrosis, and improving ovarian function (Dominici *et al.*, 2006; Uccelli *et al.*, 2008; Chen *et al.*, 2018; Rajabzadeh *et al.*, 2019; Bozorgmehr *et al.*, 2020; Na & Kim, 2020; Liao *et al.*, 2021; Cui & Jing, 2024; Kavaldzhieva *et al.*, 2025). However, these investigations primarily focused on MSC therapy alone, without systematic evaluation against current pharmacological standards. To our knowledge, no prior studies have compared BMMSC therapy directly with Dienogest, a fourth-generation progestin widely used in clinical practice for endometriosis management (Andres *et al.*, 2015; Bedaiwy *et al.*, 2017; Wu *et al.*, 2022; Muzii *et al.*, 2023). By demonstrating that BMMSC monotherapy not only suppressed IL-1β and IL-8 expression but also significantly reduced lesion size, while Dienogest failed to do so, this study provides unique preclinical evidence of BMMSCs’ superior disease-modifying and fertility-preserving potential (Iwabe *et al.*, 1998; von Wolff *et al.*, 1999; Mahnke *et al.*, 2000; Witz, 2000; Ueda *et al.*, 2002; Dominici *et al.*, 2006; Uccelli *et al.*, 2008; Khan *et al.*, 2014; Da Broi *et al.*, 2016; Giacomini *et al.*, 2017; Sanchez *et al.*, 2017; Wu *et al.*, 2017; Chen *et al.*, 2018; Ghaneialvar *et al.*, 2018; Rajabzadeh *et al.*, 2019; Schmelzer *et al.*, 2019; Bozorgmehr *et al.*, 2020; Liao *et al.*, 2021; Bian *et al.*, 2022; Műzes & Sipos, 2022; Kulesza *et al.*, 2023; Cui & Jing, 2024; Kavaldzhieva *et al.*, 2025).

This study aimed to compare the effects of BMMSCs, Dienogest, and their combination on IL-1β and IL-8 expression and lesion size in a murine endometriosis model. We hypothesized that BMMSC monotherapy would provide superior disease-modifying effects compared to Dienogest or combined therapy.

## MATERIAL AND METHODS

### Ethical Approval

This experimental study was approved by the Animal Care and Use Committee of Universitas Airlangga, Surabaya, Indonesia (Approval No. 3.KEH.145.10.2024). All procedures adhered to international standards for animal research. Humane endpoints were defined, and animals were monitored daily for weight, activity, and distress. Mice were euthanized using isoflurane overdose followed by cervical dislocation, in accordance with AVMA Guidelines (2020).

### Study period and location

The experiment was conducted between August and December 2024 at the Stem Cell Laboratory, Institute of Tropical Disease, and the Faculty of Veterinary Medicine, Universitas Airlangga, Surabaya.

### Preparation of bone marrow mesenchymal stem cells

Bone marrow aspirates were obtained from 3-month-old female mice (25-30 g). Under local anesthesia, tibial marrow was collected from 5-10 donor mice, yielding ~3 mL of aspirate, which was transferred into heparinized tubes containing an equal volume of minimum essential medium-alpha (Invitrogen, USA) and stored at 4°C until processing.

Samples were diluted with phosphate-buffered saline (PBS; Sigma, USA) and centrifuged twice at 1600 rpm for 15 min. The mononuclear cell fraction (buffy coat) was isolated by Ficoll density gradient (GE Healthcare, UK), washed with PBS, resuspended in 6 mL of complete culture medium (CCM; Invitrogen, USA), and seeded into 5 cm^2^ culture dishes. Cultures were maintained at 37°C in 5% CO₂. After 24 h, non-adherent cells were removed by washing with PBS and replaced with fresh CCM. Medium was changed every 3 days until cultures reached 60-80% confluence.

Cells were subcultured every 5 days until passage 4. Phenotypic confirmation was performed by immunofluorescence staining, requiring negativity for CD45 and positivity for CD73, CD90, and CD105, consistent with ISCT minimal criteria (Dominici *et al.*, 2006; Uccelli *et al.*, 2008; Ghaneialvar *et al.*, 2018; Schmelzer *et al.*, 2019). These markers have also been validated in previous studies characterizing MSCs in various species (Chen *et al.*, 2018; Na & Kim, 2020).

### Experimental animals

Thirty-two female BALB/c mice (3 months old, 25-30 g; Charles River, USA) were acclimatized for one week before randomization into four groups (n=8 each). Animals were housed under controlled conditions (22±2°C, 55±5% humidity, 12/12 h light-dark cycle) with free access to standard chow and water. Randomization was computer-generated, and outcome assessors were blinded. Exclusion criteria included perioperative death or failed lesion induction.

**K+:** Endometriosis control group

**P1:** BMMSC therapy (1 × 10^6^ cells in 0.2 mL PBS, i.p., day 15)

**P2:** BMMSC + Dienogest therapy (same BMMSC dose + dienogest 1 mg/kg/day p.o. from day 15-29)

**P3:** Dienogest therapy (1 mg/kg/day p.o. from day 15-29)

### Monitoring of safety and side effects

Throughout the experiment, animals were monitored daily for body weight, activity, grooming, and signs of distress (Harada *et al.*, 1999). No mortality occurred. Necropsy of major non-reproductive organs (liver, kidney, spleen) revealed no gross abnormalities, indicating that BMMSC administration did not induce systemic toxicity (Dominici *et al.*, 2006; Uccelli *et al.*, 2008).

### Establishment of the endometriosis mouse model

Endometriosis was induced following previously described protocols (Gupta *et al.*, 2008; Bedaiwy *et al.*, 2017). This murine model was selected because surgical induction by transplantation of endometrial tissue reliably reproduces the pathophysiological features of human disease, including peritoneal lesion formation, estrogen dependency, and chronic inflammatory cytokine responses (Vernon & Wilson, 1985; Bedaiwy *et al.*, 2017).

Mice received intramuscular cyclosporin A (10 mg/kg; Sandimmune, Novartis, Switzerland) and estrogen priming. A 0.1 mL suspension of human endometrial tissue was injected intraperitoneally to establish lesions. Estrogen supplementation (5.4 mg/mouse; equivalent to 10IU per 1 mg) was administered daily from days 1-5. By day 14, visible lesions had formed. BMMSCs (1×10^6^ cells/mouse) were injected intraperitoneally on day 15. All animals were sacrificed on day 29, and tissues collected for analysis. Euthanasia was performed in accordance with the protocol approved by the Institutional Animal Care and Use Committee of Universitas Airlangga (Approval No. 3.KEH.145.10.2024).

### Immunohistochemical detection of IL-1β and IL-8

Formalin-fixed, paraffin-embedded ovarian tissues were sectioned at 5 µm and deparaffinized in xylene (3 × 3 min), followed by graded ethanol rehydration (100%, 95%, 70%) and rinsing in distilled water. Endogenous peroxidase activity was blocked using peroxidase solution (27°C, 10 min). Sections were incubated in blocking serum (25°C, 10 min), then with polyclonal anti-IL-1β and anti-IL-8 primary antibodies (Bioss Antibodies) for 10 min.

After washing in PBS, slides were incubated with horseradish peroxidase-conjugated secondary antibody (25°C, 10 min), developed using diaminobenzidine (DAB, 10min), and counterstained with hematoxylin and eosin (3 min). Sections were dehydrated, mounted, and examined under light microscopy (Nikon H600L, DS-Fi2 digital camera, Nikon Image System). Brown cytoplasmic staining was interpreted as positive expression in glandular epithelial and stromal cells (Iwabe *et al.*, 1998; Mahnke *et al.*, 2000; Muzii *et al.*, 2023).

### Lesion measurement

Endometriotic lesion dimensions were evaluated using ImageJ v1.54 (NIH, USA). Lesions were photographed, calibrated against a scale bar, outlined, and measured automatically to calculate the surface area (mm^2^). Such image-based quantitative analysis is widely applied in regenerative and MSC-related studies, including wound healing models (Andres *et al.*, 2015; Wu *et al.*, 2022; Muzii *et al.*, 2023). Results were expressed as mean lesion size per group. In addition to calculating lesion size, the sample was analyzed semi-quantitatively using the IRS semi-quantitative scale, as expressed in Table 1.

**Table 1:** IRS semi-quantitative scale.

A	B
**Score 0:** No positive cells	**Score 0:** No color reaction
**Score 1:** Positive cells <10%	**Score 1:** Low color intensity
**Score 2:** Positive cells 11%-50%	**Score 2:** Medium color intensity
**Score 3:** Positive cells 51%-80%	**Score 3:** High color intensity
**Score 4:** Positive cells >80%	

IRS=Immunoreactive score.

### Statistical analysis

This study applied different analysis techniques depending on the data distribution pattern. For example, One-way analysis of variance (ANOVA) was applied to normally distributed variables. Meanwhile, if the data were not normally distributed, the Kruskal-Wallis test was performed. Following that, the study conducted a post hoc Bonferroni correction for multiple comparisons. IL-1β expression followed normal distribution and was analyzed using ANOVA, whereas IL-8 expression and lesion size were analyzed using the Kruskal-Wallis test (Harada *et al.*, 1999). The work has been reported in line with the ARRIVE guidelines 2.0.

## RESULTS

This study consisted of three stages: stem cell preparation, induction of the endometriosis model, and evaluation of BMMSC effects on IL-1β, IL-8, and lesion area. Stem cells were confirmed as mesenchymal by immunophenotyping (CD73^+^, CD90^+^, CD105^+^, and CD45^-^), and their homing capacity was verified by PKH26 luminescence (Iwabe *et al.*, 1998; Uccelli *et al.*, 2008; Andres *et al.*, 2015; Becker *et al.*, 2017; Viganò *et al.*, 2018). To validate the model, mice from all groups (K^+^-P3) were euthanized on day 14, showing established peritoneal lesions.

### Isolation and culture of bone marrow mesenchymal stem cells

**Figure 1:**
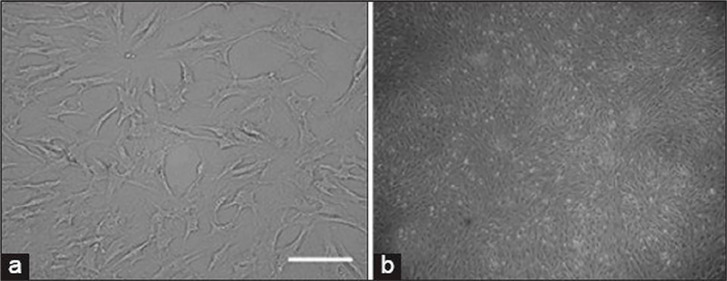
Mus musculus bone marrow mesenchymal stem cell culture. (a) Mesenchymal stem cell morphology. Cells appear to be small cell bodies (fibroblast-shaped), which are long and flattened with a large nucleus; (b) swirling pattern in passage four [inverted microscope, 40×.

Bone marrow aspirates from mouse tibiae were processed according to standard protocols in the Stem Cell Laboratory, Institute of Tropical Disease, Universitas Airlangga. Cultured cells exhibited fibroblast-like morphology, appearing elongated, flattened, and spindle-shaped with large nuclei. Subculturing was performed every 5 days until the fourth passage. By passage 4, cultures displayed the characteristic swirling growth pattern of mesenchymal stem cells (Figure 1).

### Characterization of bone marrow mesenchymal stem cells

**Figure 2:**
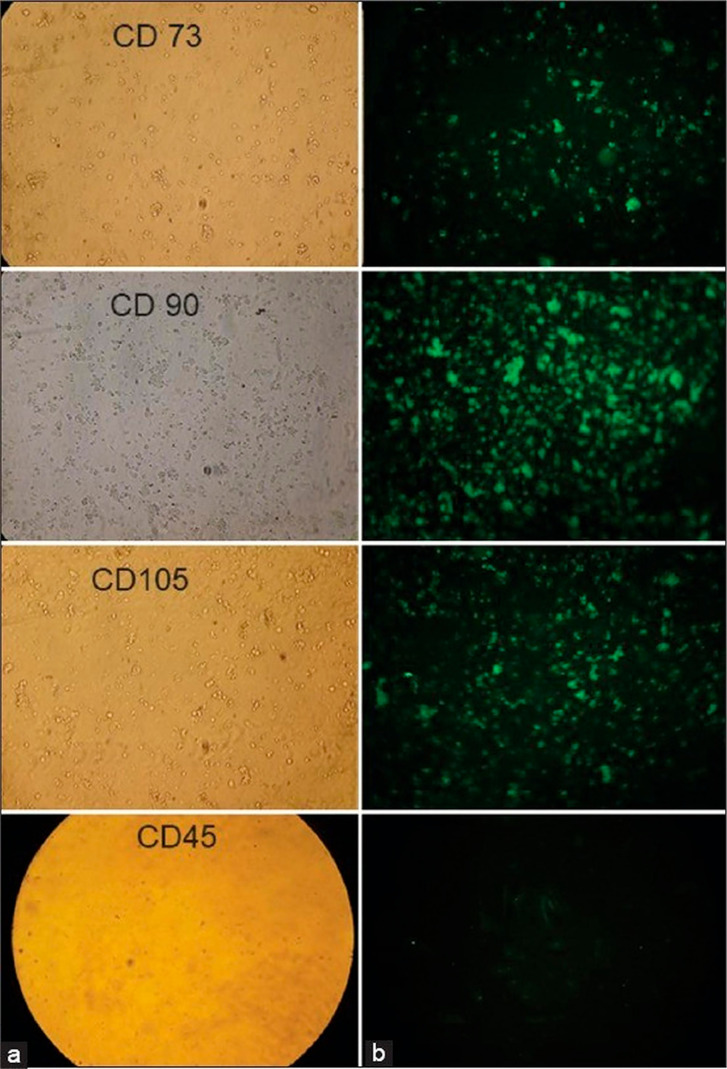
Immunohistocytochemical examination of CD 73, CD90, CD105, and CD 45. (a) Observation of bone marrow mesenchymal stem cells without fluorescence; (b) fluorescent observation of bone marrow mesenchymal stem cells (fluorescent microscope, 100×).

Stem cells developed in vitro can be characterized using both genotypic and phenotypic approaches. In this study, phenotypic identification was performed by immunocytochemistry using monoclonal antibodies conjugated with fluorescein isothiocyanate (FITC) (F3651; Sigma, St. Louis, MO). BMMSCs were confirmed by the expression of CD73, CD90, and CD105, and the absence of CD45 to exclude hematopoietic contamination (Figure 2). Immunofluorescence analysis demonstrated strong CD73 and CD90 expression, while no green luminescence was detected for CD45, consistent with the ISCT minimal criteria (Vernon & Wilson, 1985; Iwabe *et al.*, 1998; Grümmer, 2006).

**Figure 3:**
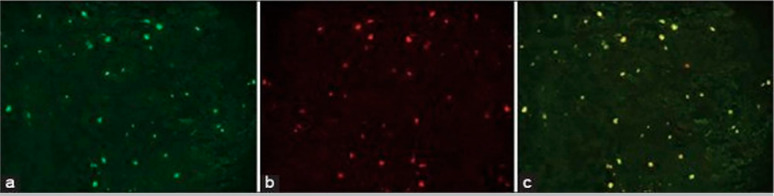
PKH26 luminescence in ovarian preparations of mice with endometriosis labeled PKH26. (a) Green filter, (b) red filter, (c) red-green filter (fluorescent microscope, 4.2×).

PKH26 fluorescence was observed in the membranes of transplanted BMMSCs within ovarian tissue (Figure 3), confirming their ability to home to the target site (Viganò *et al.*, 2018; Piriyev *et al.*, 2025).

**Figure 4:**
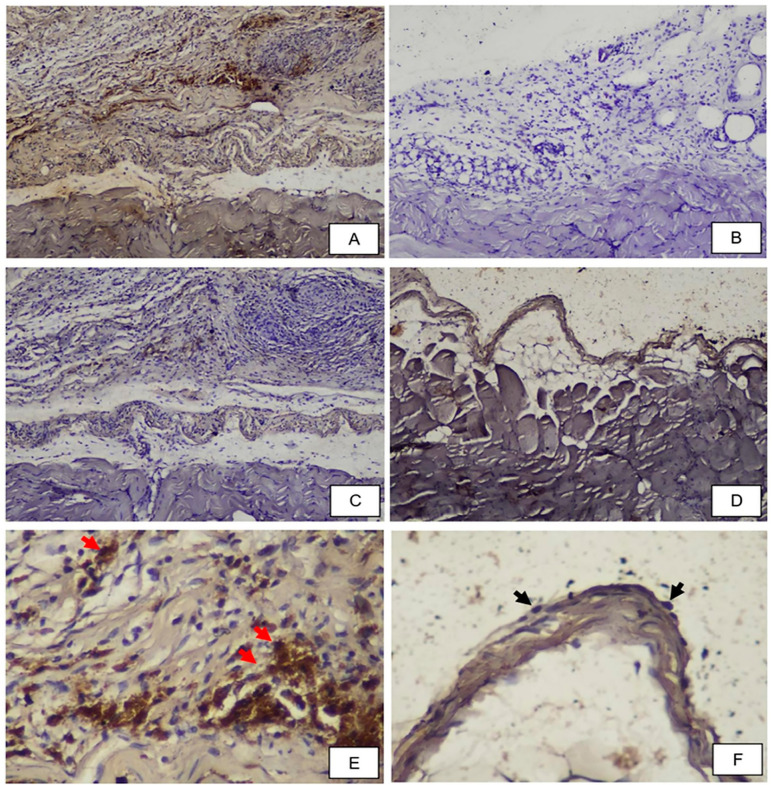
IL-1β protein expression in peritoneal endometriosis.

Figure 4 indicates that immunohistochemical staining patterns of IL-1β were observed in K+, P1, P2, and P3 groups (A, B, C, and D, respectively). Strong positive reactions were evident in the K+ group, whereas weak positive reactions were noted in the P1 group (100×). Most positively stained cells were macrophages (red arrows) (E) and mesothelial cells (black arrows) (F), both showing cytoplasmic expression of IL-1β (400×) (Iwabe *et al.*, 1998; Mahnke *et al.*, 2000; Piriyev *et al.*, 2025).

**Figure 5:**
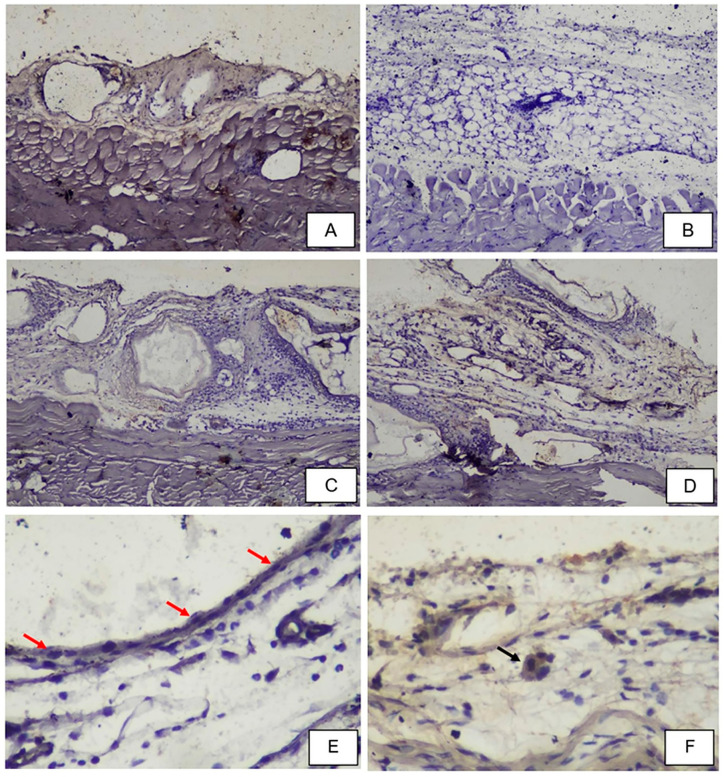
IL-8 protein expression in peritoneal endometriosis.

Figure 5 shows that the control group (K^+^) exhibited the highest IL-1β and IL-8 expression, whereas BMMSC-treated animals (P1) showed the lowest levels. Dienogest alone (P3) or in combination with BMMSCs (P2) produced some reduction but remained markedly less effective than BMMSC monotherapy (von Wolff *et al.*, 1999; Ueda *et al.*, 2002; Sanchez *et al.*, 2016; 2017; Muzii *et al.*, 2023).

### Immunohistochemical Results

Table 2 shows that the control group (K+) demonstrated the highest IL-1β and IL-8 expression, whereas BMMSC-treated animals (P1) exhibited the lowest. Dienogest alone (P3) or in combination with BMMSCs (P2) provided some reduction but were markedly less effective than BMMSC monotherapy (von Wolff *et al.*, 1999; Ueda *et al.*, 2002; Sanchez *et al.*, 2016; 2017; Muzii *et al.*, 2023).

**Table 2:** IL-1β and IL-8 expression groups.

Group	IL-1β expression [n+SD]	p value	IL-8 expression [n+SD]	p value
K+	9.05 ± 0.99	0.474	8.15 ± 0.77	0.002
P1	1.65 ± 1.21	0.121	2.50 ± 1.45	0.528
P2	7.13 ± 0.65	0.982	7.45 ± 1.02	0.163
P3	4.02 ± 1.09	0.270	4.67 ± 1.29	0.178

Values are mean ± SD. Statistical analysis by ANOVA (IL-1β) and Kruskal-Wallis test (IL-8).

Kruskal-Wallis analysis showed significant differences in IL-8 expression (H=17.236, *p*<0.001). As summarized in Table 3, the post-hoc Bonferroni confirmed that BMMSC monotherapy significantly reduced IL-8 compared with all groups (*p*=0.001), while no differences were observed among control, Dienogest, and BMMSC + Dienogest (*p*=1.000). Similarly, ANOVA revealed significant group differences in IL-1β expression (F=54.808, *p*<0.001). Both BMMSC and BMMSC + Dienogest were lower than control (*p*=0.001), with BMMSC also suppressing IL-1β more than Dienogest and the combination (*p*≤0.007). No differences were found between Dienogest and either control or BMMSC + Dienogest. Although error bars in Figures 2 and 3 appear visually similar among groups, statistical analyses confirmed significant differences. Only BMMSC monotherapy demonstrated significant reduction in IL-1β, IL-8, and lesion size compared with controls (*p*<0.05). This emphasizes that statistical outcomes, not only visual inspection, are essential for interpretation. Overall, BMMSC monotherapy provided the strongest suppression of both IL-8 and IL-1β (Mahnke *et al.*, 2000; Szukiewicz, 2022; Muzii *et al.*, 2023).

**Table 3:** Pairwise Comparison of IL-8 and IL-1**β** Expression Between Groups.

Groups	Positive Control	BMMSC	Dienogest	BMMSC+Dienogest
IL-8 Positive Control BMMSC Dienogest BMMSC + Dienogest	- 0.001 1.000 1.000	0.001 - 0.001 0.001	1.000 0.001 - 1.000	1.000 0.001 1.000 -
IL-1**β** Positive Control BMMSC Dienogest BMMSC + Dienogest	- 0.001 0.423 0.001	0.001 - 0.001 0.001	0.423 0.001 - 0.066	0.001 0.001 0.066 -

Post Hoc Bonferroni test

**Figure 6:**
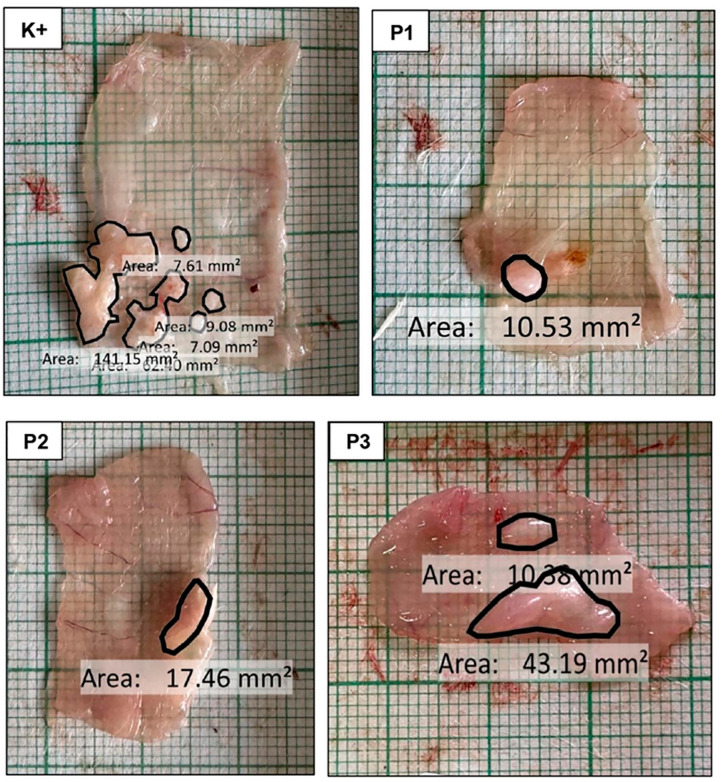
Macroscopic appearance of implant lesions.

Having identified the effectiveness of different approaches, the research evaluated the lesion area by calculating the nodule surface area using Roaster image analysis. Figure 6 compares the macroscopic appearance of implant lesions and hypervascularization of endometriosis in the peritoneal tissue of each group (K+, P1, P2, and P3).

ImageJ v1.54 (NIH, USA) analysis confirmed that BMMSC therapy was the only intervention associated with a significant reduction. The mean lesion size was 6.91 mm^2^, with 50% of BMMSC-treated mice completely free of lesions, compared to large, hypervascular lesions in the control group (mean 52.89 mm^2^, maximum 227.33 mm^2^). Neither Dienogest nor combination therapy demonstrated significant effects (Table 4).

**Table 4:** Lesion area (mm^2^) across groups.

Group	Mean ± SD [mm^2^]	Range [mm^2^]
T1 → K+ (Control)	52.89±64.72	4.49 - 227.33
T2 → P1 (BMMSC)	6.91±7.25	0 - 19.51
T3 → P2 (BMMSC + Dienogest)	18.18±19.67	0 - 64.16
T4 → P3 (Dienogest)	23.28±16.91	0 - 53.57

Values are expressed as mean ± standard deviation (SD) and range. Lesion size was analyzed using the Kruskal-Wallis test with Bonferroni post hoc.

As shown in Table 5, the Post-hoc Bonferroni after the Kruskal-Wallis test (H=10.397, *p*=0.015) showed that only BMMSC therapy reduced lesion size versus control (*p*=0.021), while Dienogest and BMMSC + Dienogest did not differ. BMMSC was the only effective treatment.

**Table 5:** Analysis of differences in endometriotic lesion size among groups.

Comparison	p-value	Significance
Positive Control vs BMMSC	0.021	Significant
Positive Control vs Dienogest	1.000	Not significant
Positive Control vs BMMSC + Dienogest	0.711	Not significant
BMMSC vs Dienogest	0.234	Not significant
BMMSC vs BMMSC + Dienogest	1.000	Not significant
Dienogest vs BMMSC + Dienogest	1.000	Not significant

Post-hoc Bonferroni test following Kruskal-Wallis (H=10.397, *p*=0.01)].

## DISCUSSION

The BMMSCs characterized in this study exhibited the expected immunophenotypic profile of mesenchymal stem cells, expressing CD73, CD90, and CD105 while lacking CD45, in accordance with the International Society for Cellular Therapy (ISCT) criteria (Iwabe *et al.*, 1998). This confirmed that the transplanted cells were genuine BMMSCs with immunomodulatory and regenerative potential. Moreover, PKH26 fluorescence verified their ability to migrate and engraft into ovarian tissue, demonstrating successful homing (Piriyev *et al.*, 2025).

Endometriosis is strongly associated with chronic pelvic inflammation, leading to elevated peritoneal cytokine concentrations that disrupt folliculogenesis (Mahnke *et al.*, 2000; Muzii *et al.*, 2023). In the present study, immunohistochemistry revealed high expression of IL-1β and IL-8 in glandular epithelial and stromal cells of the control group, whereas BMMSC treatment markedly suppressed both cytokines. These findings are consistent with earlier evidence implicating IL-1β and IL-8 in lesion proliferation, angiogenesis, fibrotic progression, and reduced oocyte competence (Mahnke *et al.*, 2000; Szukiewicz, 2022; Piriyev *et al.*, 2025).

BMMSCs are recognized for their capacity to downregulate inflammatory mediators through paracrine mechanisms, including secretion of IL-10, prostaglandin E₂, and TGF-β, as well as inhibition of NF-κB signaling (Xu *et al.*, 2025). Such mechanisms likely account for the robust cytokine suppression observed in the BMMSC group.

### Role of inflammatory cytokines in endometriosis

Endometriosis is characterized by a chronic inflammatory peritoneal environment. Elevated levels of IL-1β, IL-8, and TNF-α are consistently detected in the peritoneal and follicular fluid of affected women, where they contribute to angiogenesis, fibrotic remodeling, granulosa cell apoptosis, and impaired steroidogenesis (Iwabe *et al.*, 1998; Mahnke *et al.*, 2000; Witz, 2000; Ueda *et al.*, 2002; Khan *et al.*, 2014; Da Broi *et al.*, 2016; Giacomini *et al.*, 2017; Sanchez *et al.*, 2017; Wu *et al.*, 2017). These cytokines have also been implicated in poor oocyte competence and reduced IVF success rates (Barnhart *et al.*, 2002; Omland *et al.*, 2005; Gupta *et al.*, 2008; Da Broi *et al.*, 2016; Giacomini *et al.*, 2017; Sanchez *et al.*, 2017; Wu *et al.*, 2017; Viganò *et al.*, 2018). Our findings confirm that IL-1β and IL-8 expression were highest in untreated controls, reinforcing their central role in disease persistence.

### Limitations of hormonal therapy

Hormonal therapies, including Dienogest, primarily act by suppressing ovulation and inducing endometrial atrophy (Andres *et al.*, 2015; Bedaiwy *et al.*, 2017; Wu *et al.*, 2022; Muzii *et al.*, 2023). Although they provide symptomatic relief, they do not directly modulate the inflammatory microenvironment, and recurrence is common once treatment is discontinued (Andres *et al.*, 2015; Becker *et al.*, 2017; Bedaiwy *et al.*, 2017; Szukiewicz, 2022; Wu *et al.*, 2022; Muzii *et al.*, 2023; Piriyev *et al.*, 2025). In the present study, Dienogest did not significantly reduce IL-1β, IL-8, or lesion size compared with controls, consistent with clinical evidence of incomplete efficacy (Wu *et al.*, 2022; Muzii *et al.*, 2023; Piriyev *et al.*, 2025).

### Mechanisms of BMMSC therapy

BMMSCs exert their therapeutic effects primarily through paracrine signaling, secreting prostaglandin E₂, IL-10, and TGF-β, and inhibiting NF-κB activation (Dominici *et al.*, 2006; Uccelli *et al.*, 2008; Chen *et al.*, 2018; Ghaneialvar *et al.*, 2018; Schmelzer *et al.*, 2019; Bian *et al.*, 2022; Műzes & Sipos, 2022; Kulesza *et al.*, 2023). These mechanisms suppress inflammatory cascades and modulate immune cell activity. In addition, BMMSC-derived extracellular vesicles and secretomes protect granulosa cells from apoptosis, reduce oxidative stress, and promote follicular repair (Chen *et al.*, 2018; Rajabzadeh *et al.*, 2019; Bozorgmehr *et al.*, 2020; Na & Kim, 2020; Liao *et al.*, 2021; Cui & Jing, 2024; Kavaldzhieva *et al.*, 2025). In the present study, BMMSC monotherapy achieved the most pronounced suppression of IL-1β and IL-8 and significantly reduced lesion size, consistent with their established immunomodulatory and regenerative roles.

### Lack of synergy with Dienogest

The absence of synergistic effects between BMMSCs and Dienogest may be attributed to potential interactions between hormonal suppression and MSC regenerative pathways. Hormonal therapy could interfere with MSC homing or paracrine activity, thereby diminishing their therapeutic efficacy. Similar observations have been reported in studies where immunomodulatory therapies showed reduced effects when combined with hormonal suppression^20-22^.

### Clinical implications

BMMSC therapy offers several potential advantages over conventional management. By directly targeting inflammation and tissue remodeling, BMMSCs may provide disease-modifying and fertility-preserving benefits, which are particularly valuable for women seeking pregnancy (Barnhart *et al.*, 2002; Omland *et al.*, 2005; Da Broi *et al.*, 2016; Sanchez *et al.*, 2016; 2017; Giacomini *et al.*, 2017; Wu *et al.*, 2017; Rajabzadeh *et al.*, 2019; Bozorgmehr *et al.*, 2020; Liao *et al.*, 2021; Cui & Jing, 2024; Kavaldzhieva *et al.*, 2025).

### Study limitations

Although this study offers important findings, several limitations must be acknowledged. First, the observation period was relatively short (14 days post-treatment). Second, angiogenic and fibrotic markers such as VEGF and TGF-β were not evaluated, despite their central roles in endometriosis progression lin (Xu *et al.*, 2025). Finally, murine models cannot fully reproduce the complexity of human disease (Vernon & Wilson, 1985; Grümmer, 2006). Future studies should address these limitations through longer observation periods, broader biomarker assessment, and eventual translation into clinical trials.

Mechanistically, the therapeutic advantage of BMMSCs is attributed primarily to paracrine effects rather than cellular differentiation. Their secretome has been shown to suppress inflammation, inhibit angiogenesis, alleviate oxidative stress, and prevent granulosa cell apoptosis. By reshaping the peritoneal microenvironment, BMMSCs disrupt the self-sustaining cycle of cytokine release and lesion survival. The lack of synergy between BMMSCs and Dienogest may be explained by hormonal suppression interfering with BMMSC regenerative and immunomodulatory pathways (Dominici *et al.*, 2006; Uccelli *et al.*, 2008; Chen *et al.*, 2018; Na & Kim, 2020).

### Comparison with previous studies and novelty

Previous studies have reported beneficial effects of mesenchymal stem cells in endometriosis models (Chen *et al.*, 2018; Rajabzadeh *et al.*, 2019; Bozorgmehr *et al.*, 2020; Na & Kim, 2020; Liao *et al.*, 2021; Cui & Jing, 2024; Kavaldzhieva *et al.*, 2025). However, these investigations generally focused on MSC transplantation alone without direct comparison to standard pharmacological therapy. To our knowledge, this is the first study to perform a head-to-head evaluation of BMMSCs, Dienogest, and their combination in a murine endometriosis model. The novelty of our findings lies in demonstrating that BMMSC monotherapy not only suppressed pro-inflammatory cytokines (IL-1β and IL-8) but also significantly reduced lesion size, whereas Dienogest alone did not. This provides unique preclinical evidence of the superior disease-modifying potential of BMMSCs compared with standard care.

From a clinical perspective, BMMSC therapy may represent a disease-modifying and fertility-preserving approach for endometriosis. The strong suppression of IL-1β and IL-8, together with lesion regression, provides compelling preclinical evidence. Nonetheless, several limitations must be acknowledged. The observation period was relatively short, angiogenic and fibrotic markers such as VEGF and TGF-β were not assessed (Xu *et al.*, 2025), and murine models cannot fully recapitulate the complexity of human disease (Vernon & Wilson, 1985; Grümmer, 2006). Another limitation is that BMMSC characterization was performed by immunofluorescence (CD73^+^, CD90^+^, CD105^+^, CD45^+^) but was not validated by flow cytometry due to facility limitations. Future studies should include longer-term evaluation, broader biomarker panels, and flow cytometric profiling to comply fully with ISCT recommendations (Dominici *et al.*, 2006; Chen *et al.*, 2018; Na & Kim, 2020).

From a translational perspective, BMMSC therapy holds promise as a fertility-preserving and disease-modifying approach for endometriosis. The strong suppression of IL-1β and IL-8, together with lesion regression, suggests potential applications in women seeking pregnancy while minimizing recurrence. Future studies should extend the observation period, evaluate angiogenic and fibrotic markers such as VEGF and TGF-β (Xu *et al.*, 2025), and advance toward clinical trials to determine long-term efficacy and safety. To our knowledge, this is the first study to directly compare BMMSCs with standard hormonal therapy in endometriosis models, reinforcing their potential as a regenerative therapeutic strategy.

## CONCLUSION

BMMSC monotherapy demonstrated superior efficacy compared with Dienogest or combination therapy in suppressing IL-1β and IL-8 expression and in reducing lesion size in murine endometriosis models. These findings underscore BMMSCs as a promising regenerative, disease-modifying, and fertility-preserving therapeutic strategy for the management of endometriosis (Dominici *et al.*, 2006; Uccelli *et al.*, 2008; Chen *et al.*, 2018; Ghaneialvar *et al.*, 2018; Rajabzadeh *et al.*, 2019; Schmelzer *et al.*, 2019; Bozorgmehr *et al.*, 2020; Liao *et al.*, 2021; Bian *et al.*, 2022; Műzes & Sipos, 2022; Kulesza *et al.*, 2023; Cui & Jing, 2024; Kavaldzhieva *et al.*, 2025).
